# Integrative Radiogenomics Using MRI Radiomics and Microarray Gene Expression Analysis to Predict Pathological Complete Response in Patients with Breast Cancer Undergoing Neoadjuvant Chemotherapy

**DOI:** 10.7759/cureus.86287

**Published:** 2025-06-18

**Authors:** Soya Oda, Yukiko Tokuda, Yuki Suzuki, Masahiro Yanagawa, Yoshiaki Sota, Yasuto Naoi, Yuichi Motoyama, Eiichi Morii, Kenzo Shimazu, Shoji Kido, Noriyuki Tomiyama, Masatoshi Hori

**Affiliations:** 1 Department of Artificial Intelligence in Diagnostic Radiology, The University of Osaka Graduate School of Medicine, Suita, JPN; 2 Department of Interventional and Diagnostic Radiology, The University of Osaka Graduate School of Medicine, Suita, JPN; 3 Department of Breast and Endocrine Surgery, The University of Osaka Graduate School of Medicine, Suita, JPN; 4 Endocrine and Breast Surgery, Kyoto Prefectural University of Medicine, Kyoto, JPN; 5 Department of Pathology, The University of Osaka Graduate School of Medicine, Suita, JPN

**Keywords:** breast neoplasms, magnetic resonance imaging, neoadjuvant chemotherapy, radiogenomics, radiomics

## Abstract

Objectives: Given the variable pathological complete response (pCR) rate of neoadjuvant chemotherapy (NAC) in patients with breast cancer, identifying predictive markers is crucial. This study evaluated the predictive accuracy of three machine learning-based models: (1) radiomics using MRI features; (2) genomics based on DNA microarray data; and (3) radiogenomics integrating both MRI and microarray data to predict pCR after NAC across all breast cancer subtypes. This study aimed to determine which model provides the most precise non-invasive prediction by utilizing a consistent dataset and analytical pipeline.

Methods: In this retrospective study, 112 patients with breast cancer who underwent DNA microarray analysis and dynamic contrast-enhanced MRI before receiving NAC at a single institution between July 2006 and November 2016 were classified into pCR (N = 21) and non-pCR (N = 91) groups. The prediction accuracy of pCR after NAC was evaluated for three models using repeated stratified nested cross-validation (CV). Model performance was assessed by the area under the receiver operating characteristic curve (ROC-AUC), and statistical significance was tested using DeLong’s test.

Results: Among the 112 patients, the radiogenomics model yielded an AUC of 0.607 (95% confidence interval (CI): 0.438-0.758), outperforming both the radiomics (AUC 0.563, 95% CI: 0.410-0.718) and the genomics (AUC 0.559, 95% CI: 0.379-0.722) models. However, this improvement was not statistically significant (p>0.05).

Conclusion: Machine learning-based radiogenomics, which combines MRI features and DNA microarray data, improved the accuracy of pCR prediction after NAC, although the improvement was not statistically significant. These findings suggest the potential utility of radiogenomics as a non-invasive tool to support treatment decision-making in patients undergoing NAC.

## Introduction

Breast cancer is a significant global health concern, with both high morbidity and mortality rates among women [1,2]. Its diverse biochemical natures lead to varied therapy responses [3]. Therefore, the need for personalized treatment is increasing.

Neoadjuvant chemotherapy (NAC) is crucial for breast cancer treatment, aiming for a pathological complete response (pCR) and favorable outcomes [4]. However, some patients have shown resistance, making it essential to provide alternative treatment options. Therefore, identifying patients unlikely to benefit from NAC is pivotal [5].

MRI is important for evaluating the pCR before and after NAC in patients with breast cancer [6,7]. However, its accuracy in predicting the treatment response varies [8-11]. Several studies have explored the potential of radiomics in predicting pCR [12-15]. Fan et al. identified 12 radiomic features associated with tumor response to NAC using 57 pre-NAC MRI scans [16]. Similarly, Zheng et al. showed that a nomogram combining radiomic signatures from the intratumoral and peritumoral regions with clinical characteristics could accurately predict pCR after NAC [17]. Several researchers have reported the value of radiomics in predicting pCR in patients with breast cancer using MRI [16,18-23]. Reported area under the curve (AUC) values have ranged from 0.64 to 0.78 [18,19,21]. Conversely, gene expression analysis methods have developed over the past two decades, enabling the construction of recurrence prediction models based on gene expression in breast cancer tissues. However, their accuracy is reportedly insufficient to reliably identify patients who are less likely to achieve pCR, complicating the determination of NAC indications based solely on this technique [24]. Recent studies have begun to explore the integration of imaging and genomic data, an approach known as radiogenomics, to improve prediction of treatment response in breast cancer. For example, radiogenomic models have shown promising results in predicting pCR and prognosis, particularly in patients with triple-negative breast cancer [25,26]. However, comprehensive integration of MRI features and microarray-based gene expression data across all breast cancer subtypes remains limited. Given the limitations of MRI and gene expression analysis when used independently, we hypothesized that integrating machine learning with both MRI and microarray data (radiogenomics) could compensate for the weaknesses of each modality and improve the accuracy of pCR prediction after NAC. This approach aims to address a key clinical challenge, that is, identifying likely responders to NAC more reliably, by combining complementary information from imaging and genomic data.

This study aimed to investigate the efficacy of an integrated approach, termed radiogenomics, for predicting pCR after NAC. We hope to identify the most reliable and robust method for predicting the response to NAC, which can inform future therapeutic strategies for personalized therapy in patients with breast cancer.

## Materials and methods

Study participants

**Figure 1:**
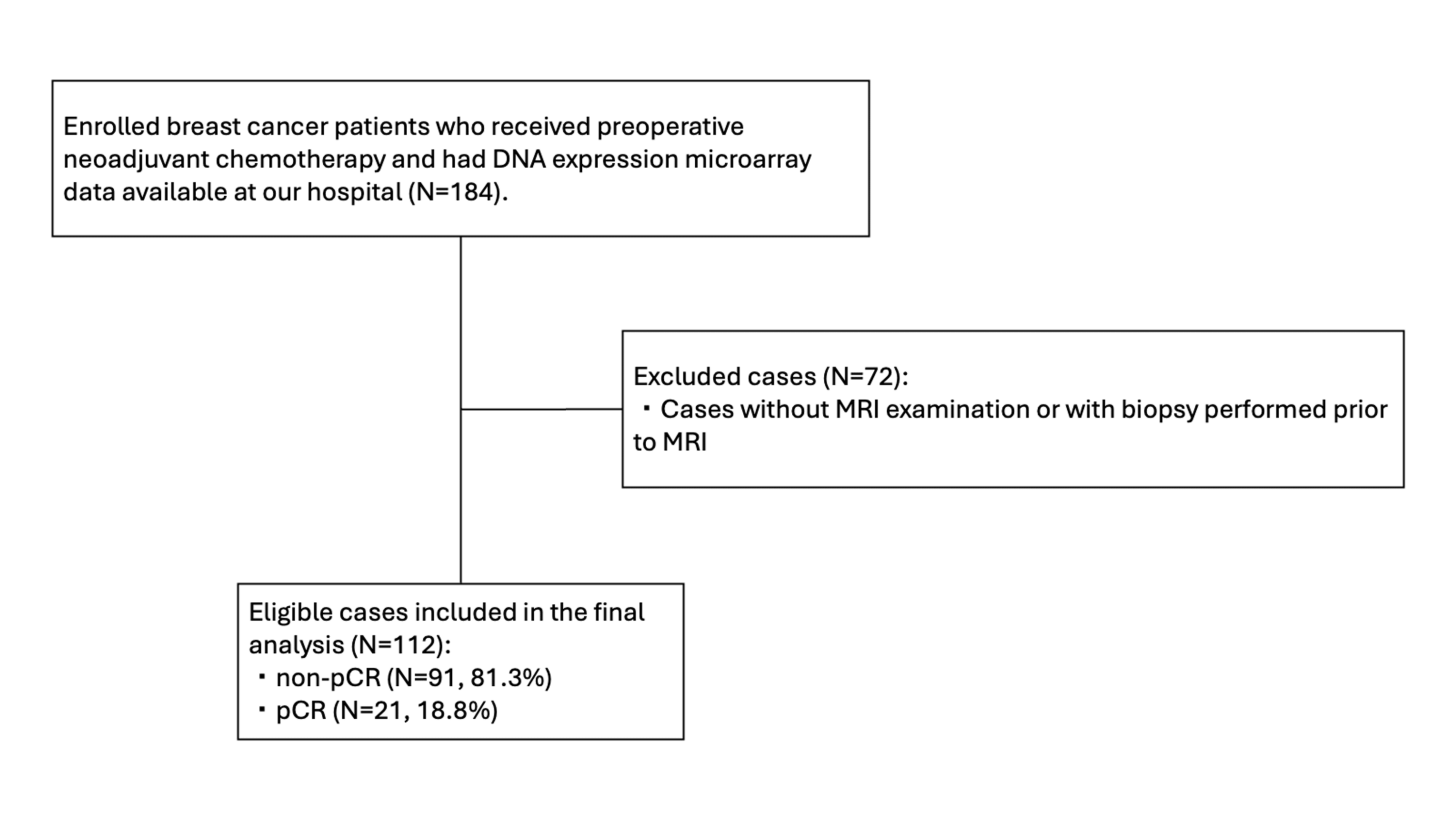
Flowchart depicting patient selection From 2002 to 2016, 184 patients with breast cancer who received preoperative NAC and had DNA expression microarray data available were enrolled. Patients were excluded if they did not undergo MRI examination or if a biopsy was performed prior to MRI (N = 72/184, 39.1%). The final analysis included 112 eligible patients, classified into pCR (N = 21/112, 18.8%) and non-pCR (N = 91/112, 81.3%). Data are presented as N (number of patients). NAC: Neoadjuvant chemotherapy; pCR: Pathological complete response

This study was conducted with the approval of the Institutional Review Board (IRB number: 15567-10), and the requirement for informed consent was waived. Prior to NAC, all patients underwent vacuum-assisted biopsy for histological examination. This retrospective study was conducted at our hospital between 2002 and 2016. The inclusion criteria were patients who underwent NAC and had available microarray data (184), as shown in Figure 1. We excluded 72 patients due to the absence of an MRI examination, lack of preoperative contrast-enhanced MRI, or biopsy performed prior to MRI. This resulted in 112 patients eligible for the final analysis. These patients underwent gene expression analyses before NAC, and their breast cancer subtypes and other relevant characteristics were documented in their health records, as shown in Table 1. Among the enrolled patients, 91 (81.3%) were identified as non-pCR, while the remaining 21 (18.8%) achieved pCR after NAC. These patients received NAC as previously described with some modifications [27,28].

**Table 1:** Patient characteristics Data are presented as mean±SD for continuous variables and N (%) for categorical variables. Statistical significance was determined using Welch’s two-sample t-test (for Age), Pearson’s chi-squared test (for Ki-67, T-stage, N-stage, and Breast Cancer Subtype), and Fisher's exact test (for Histological Grade, where cell counts were low). The corresponding test statistic values (t-value or χ²-value) are included in the table. P-value <0.05 was considered statistically significant. TNM classification based on the 15th edition pCR: Pathological complete response; HER2: Human epidermal growth factor receptor 2

Characteristics		Non-pCR, N = 91	pCR, N = 21	p-value
Age		52.8 (11.6)	58.5 (9.8)	0.026
Ki-67 (≥20%)				0.15
	Negative	40 (44%)	5 (24%)	
	Positive	51 (56%)	16 (76%)	
Histological Grade				0.3
	1	17 (19%)	1 (4.8%)	
	2	49 (54%)	12 (57%)	
	3	25 (27%)	8 (38%)	
T-stage				0.8
	1	5 (5.5%)	1 (4.8%)	
	2	70 (77%)	15 (71%)	
	3	9 (9.9%)	2 (9.5%)	
	4	7 (7.7%)	3 (14%)	
N-stage				0.2
	0	26 (29%)	5 (24%)	
	1	65 (71%)	15 (71%)	
	2	0 (0%)	1 (4.8%)	
Breast Cancer Subtype				<0.001
	HER2 enriched	22 (24%)	13 (62%)	
	Luminal A	29 (32%)	1 (4.8%)	
	Luminal B	28 (31%)	1 (4.8%)	
	Triple negative	12 (13%)	6 (29%)	

The majority of patients (102/112, 91.1%) received weekly paclitaxel treatment followed by fluorouracil, epirubicin, and cyclophosphamide 75 (FEC75) chemotherapy. A smaller number (1/112, 0.9%) received weekly paclitaxel followed by FEC74. Additionally, patients with human epidermal growth factor receptor 2 (HER2)-positive breast cancer (9/112, 8.0%) received weekly paclitaxel combined with trastuzumab, followed by FEC75. The FEC portion of chemotherapy was sometimes modified or interrupted according to individual patient responses and tolerability.

It's important to note that in some cases, the FEC portion of the regimen was interrupted or modified based on individual patient responses and tolerability.

Histological evaluation of response to chemotherapy

Surgical specimens were cut into 5-mm slices, and hematoxylin and eosin-stained 3-μm sections were prepared to determine the presence or absence of tumor cells. Pathological diagnoses were performed by a pathologist with eight years of experience. The complete disappearance of invasive tumor cells in the breast and negative lymph nodes was defined as pCR, regardless of the presence or absence of ductal carcinoma in situ in the breast [29].

Microarray features

As described in previous studies , RNA was extracted using TRIzol (Invitrogen, USA) from core-needle tumor biopsy samples obtained using the Mammotome (Mammotome 8G; HH Ethicon Endosurgery/Johnson and Johnson Company, USA) and analyzed using a DNA microarray (Human Genome U133 plus 2.0 Array; Affymetrix, USA) [27,28]. The presence of tumor cells in these samples was confirmed histologically in adjacent tumor biopsy samples. The extracted RNA (50 ng) was subjected to second-strand combinational DNA (cDNA) generation and amplification using random primers (WT-Ovation FFPE RNA Amplification System V2; NuGEN, USA). The amplified cDNA was then biotinylated, fragmented using the FL-Ovation cDNA Biotin Module V2 (NuGEN), and hybridized to the Affymetrix GeneChip Human Genome U133 Plus 2.0 Array (Affymetrix) overnight (17 h) according to the manufacturer’s protocol. After hybridization, the DNA microarray was stained for fluorescence using a GeneChip Fluidics Station 450 (Affymetrix) and scanned using a Scanner 3000 (Affymetrix). Microarray analysis of each case yielded 54,675 features.

MRI protocol

Before NAC, patients underwent MRI using a 1.5T unit (Signa EXCITE HD EchoSpeed Plus; GE Healthcare, USA) equipped with a 4-channel breast-array coil. The imaging protocol included one precontrast and four postcontrast-enhanced volumetric series captured at 94-second intervals to facilitate dynamic sequence analysis. Gadopentetate dimeglumine (Magnevist; Bayer, Japan) at a dose of 0.1 mmol/kg body weight was administered intravenously at a flow rate of 2 mL/s, followed by a 20 mL saline flush. The k-space's sequential view order was averaged over the scan time.

We used a T1-weighted fat-saturated three-dimensional gradient-echo sequence (Volume Imaging for Breast Assessment (VIBRANT); GE Healthcare) with the following parameters for the sagittal bilateral protocol: repetition time (TR) ranging from 6.3-6.5 ms; echo time (TE) ranging between 3.1-3.2 ms; in-plane resolution pixel sizes of 0.78 mm, without spacing between sections; flip angle of 10°; and a slice thickness of 2.0-2.6 mm in the slice direction. Additional parameters included a field of view of 20-22 cm and an acquisition matrix of 256×160.

MRI features extraction

For radiomic feature extraction, annotations were based on images captured 90 s after contrast in dynamic contrast-enhanced MRI. In cases with multiple tumors, the largest tumor was selected for detailed annotation. This task was manually performed by three radiologists from the radiology department with 2, 19, and 25 years of experience. During annotation, special attention was given to identifying and manually excluding areas of intratumoral hemorrhage. This segmentation, avoiding the hemorrhagic regions, defines the tumor volume of interest (VOI) for each patient.

Using PyRadiomics (version 2.2.0; https://pypi.org/project/pyradiomics/2.2.0/), images were standardized, and 1,132 radiomic features were extracted from the VOI identified for each patient. To ensure reproducibility, feature extraction was performed using settings from the official PyRadiomics YAML configuration file for MRI data with an approximately 3-mm slice thickness (https://github.com/AIM-Harvard/pyradiomics/blob/39aaa77e588ea3539a2b081b880d84f19ff17e91/examples/exampleSettings/exampleMR_3mm.yaml). This file provides a standardized set of parameters for image preprocessing, including normalization and resampling, as well as defining the specific feature classes to be extracted.

Features were extracted from both original and processed images with Laplacian of Gaussian (LoG) filters with sigma values of 2.0, 3.0, 4.0, and 5.0, and wavelet filters (resulting in eight wavelet decompositions). The extracted features included morphological (14), histogram (234), and texture-based (884) features. Morphological features were calculated from segmented images, whereas histogram-based and textural features were calculated from both original and filtered images.

The images were normalized to a scale of 100 and resampled to a voxel size of 2 mm × 2 mm × 2 mm using B-spline interpolation. A bin width of five was used for image discretization. A voxel array shift of 300 was applied to ensure that most voxel values were positive. For further details on the parameter settings, please refer to the configuration file provided at the URL.

Construction of machine learning for the predictive model for pCR after NAC

We comprehensively evaluated the performance of three distinct models in predicting pCR after NAC in patients with breast cancer. The models are based on different datasets: (1) radiomics using MRI features; (2) genomics using DNA microarray data; and (3) radiogenomics integrating MRI features and microarray data. Each model's effectiveness was quantified using the AUC, and the optimal sensitivity and specificity values were determined using the Youden index.

Machine-learning model construction and evaluation

We constructed machine-learning models to predict pCR after NAC and assessed their accuracy using a repeated stratified nested cross-validation (CV) approach. Regarding feature selection, for the radiomics model and the radiomics component of the radiogenomics model, all 1,132 features extracted via PyRadiomics were initially included, with the machine-learning algorithm itself performing implicit feature weighting and selection through its training process. For the genomics model and the genomics component of the radiogenomics model, microarray data were first filtered to retain genes with a detection p-value <0.01 in over 80% of cases; this step aimed to remove unreliably measured or low-expressed genes. If the number of remaining genes after this filtering exceeded 1,000, a random subset of 1,000 genes was selected. This random selection was performed to reduce the dimensionality of the feature space, thereby mitigating the risk of overfitting, particularly given our sample size, and to ensure computational tractability for the subsequent modeling steps, while still aiming to capture a diverse representation of genomic information. The Random Forest classifier was chosen as the specific machine-learning algorithm for developing all predictive models. The rationale for selecting Random Forest includes its demonstrated robustness in handling high-dimensional datasets (common in radiomics and genomics), its ability to model complex non-linear relationships between features and outcomes, its inherent capability to provide feature importance scores, and its relatively good performance and reduced susceptibility to overfitting compared to some other algorithms, especially in studies with limited sample sizes. Furthermore, Random Forest has been widely and successfully applied in similar biomedical prediction tasks, including breast cancer outcome prediction. We employed a five-fold stratified CV for both the outer and inner folds, which was repeated five times for the outer folds, where hyperparameters were tuned in the inner folds and prediction accuracies were evaluated on the data in the outer folds.

Our study aimed to compare the prediction accuracies of three distinct models: (1) radiomics using MRI features; (2) genomics using microarray data; and (3) radiogenomics combining microarray and MRI features. The pipeline of algorithms used in this study is summarized in Table 2.

**Table 2:** Overview of the machine learning analytical pipeline used for pCR prediction Procedures are systematically listed, outlining key steps from data preprocessing through feature selection, model training, performance assessment, and feature importance analysis. pCR: Pathological complete response; NAC: Neoadjuvant chemotherapy; ROC-AUC: Area under the receiver operating characteristic curve

Step	Procedure	Description
1	Data preprocessing and feature selection	Radiomic: All 1,132 features from PyRadiomics included. Genomic: Genes filtered (detection p<0.01 in >80% of cases); if >1,000 genes, randomly selected 1,000 (to reduce dimensionality, prevent overfitting, and ensure efficiency).
2	Classifier selection	Random Forest classifier used to predict pCR after NAC.
3	Hyperparameter tuning	Randomized search (RandomizedSearchCV) conducted with inner stratified 5-fold CV to optimize parameters: n_estimators (100–500), max_depth (None, 10–40), max_features (auto, sqrt, log2), and min_samples_split (2, 5, 10).
4	Model training and evaluation	Models trained using optimal hyperparameters derived from inner CV. Predictive accuracy validated using outer stratified 5-fold CV repeated five times.
5	Performance assessment	Model performance assessed by ROC-AUC. Sensitivity and specificity determined using Youden index.
6	Feature importance analysis	Importance scores calculated by mean decrease in impurity (Gini importance). Top 30 features identified for each model (MRI, microarray, MRI+microarray).

Statistics

All statistical analysis were performed using R software (version 4.3; https://www.r-project.org/), utilizing specific packages from the Bioconductor (version 3.18; https://www.bioconductor.org/) for microarray data processing and analysis. For comparisons of patient characteristics between the pCR and non-pCR groups, tests were selected based on the data type. Welch’s two-sample t-test was used for continuous variables, as it provides a robust comparison without assuming equal variances between groups. For categorical variables, the standard Pearson’s chi-squared test was applied. As described in Table 1, Fisher's exact test was used as an alternative in cases of low expected cell counts to maintain accuracy. All feature selections and machine-learning algorithms were performed using Python (version 3.10.0; https://www.python.org/) with the following key libraries: scikit-learn (version 1.5.1) for machine-learning tasks; pandas (version 2.2.2) for data handling; NumPy (version 1.26.4) for numerical operations; SciPy (version 1.13.1) for statistical tests; and Matplotlib (version 3.9.2) for visualization. R was also used to calculate descriptive statistics, expressed as the mean ± standard deviation. Each model's receiver operating characteristic (ROC) curves and the area under the ROC curve (ROC-AUC) were calculated. Comparisons of AUCs between the different predictive models were performed using DeLong’s test to assess statistical significance. P-value <0.05 was considered to indicate a statistically significant difference.

## Results

Radiomics model (MRI features)

The radiomics model, which utilized MRI features, demonstrated an AUC of 0.563 (95% confidence interval (CI) 0.410-0.718), indicating a moderate ability to differentiate between pCR and non-pCR patients. The sensitivity and specificity of the model were 0.524 and 0.714, respectively.

Genomics model (DNA microarray data)

The genomics model, based on DNA microarray data, showed a performance with an AUC of 0.559 (95% CI 0.379-0.722). This model showed a lower sensitivity of 0.571 but a higher specificity of 0.681 than the radiomics model. The genomic approach highlighted certain genomic features as key predictors of pCR.

Radiogenomics model (integration of MRI and microarray data)

The radiogenomics model, which integrated both MRI and microarray data, produced the most significant results. This model achieved the highest AUC of 0.607 (95% CI, 0.438-0.758), along with a sensitivity of 0.619 and a specificity of 0.681. Termination of radiomic and genomic features resulted in a more comprehensive analysis, leading to improved prediction of pCR. The p-values for comparisons between the models were as follows: MRI (radiomics) vs. microarray (genomics), p = 0.973; MRI vs. MRI+microarray (radiogenomics), p = 0.644; and microarray vs. MRI+microarray, p = 0.232.

A detailed comparison of these performance metrics is presented in Table 3. Although the differences in AUC between models were not statistically significant, the radiogenomics model showed a numerically higher AUC than either the radiomics or genomics models, with absolute differences of 0.044 and 0.048, respectively. These effect sizes are modest; however, even small improvements in predictive accuracy may be clinically meaningful, particularly in supporting treatment decisions where reliable prediction of pCR could inform the use or avoidance of NAC.

**Table 3:** Performance metrics for the machine-learning classifier Data are represented as N (%), unless otherwise specified. The AUC is presented with 95% CI. Sensitivity and specificity were calculated based on optimal thresholds determined using the Youden index. The chi-squared (χ²) test was used to compare sensitivities and specificities between the radiomics (reference) model and other models. P-value <0.05 was considered statistically significant. AUC: Area under the curve; CI: Confidence interval

Method	AUC (95% CI)	Sensitivity (%)	Specificity (%)	Test statistic	p-value
Radiomics Model	0.563 (0.410–0.718)	11/21 (52.4%)	65/91 (71.4%)	–	Reference
Genomics Model	0.559 (0.379–0.722)	12/21 (57.1%)	62/91 (68.1%)	χ² = 0.001	0.973
Radiogenomics Model	0.607 (0.438–0.758)	13/21 (61.9%)	62/91 (68.1%)	χ² = 0.214	0.644

**Figure 2:**
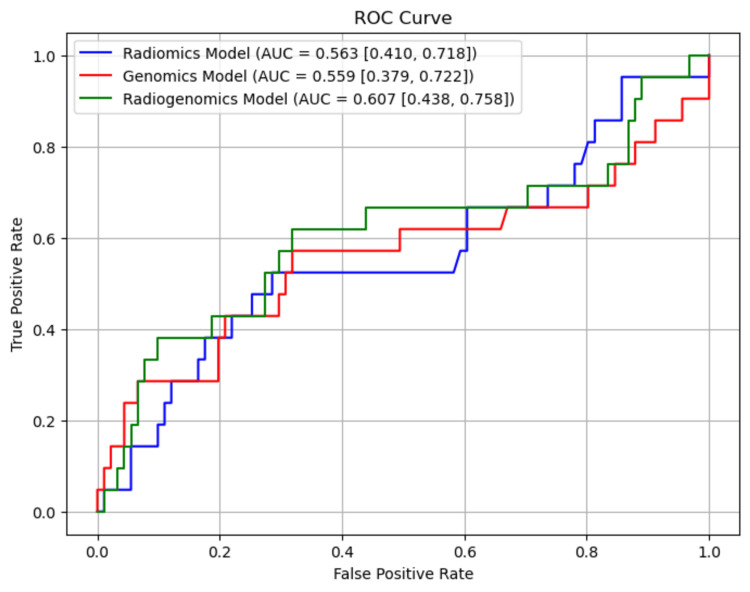
ROC curves for the prediction of pCR according to radiomics (MRI), genomics (microarray), and radiogenomics (MRI+microarray) models The ROC curves illustrate the models' ability to discriminate between (pCR and non-pCR groups (N = 112). Data are presented with AUC values and 95% CI. An AUC closer to 1 indicates better model performance. Statistical comparison between models was performed using DeLong’s test. P-value <0.05 was considered statistically significant. Although statistical significance was not achieved, the radiogenomics model showed numerically higher AUCs compared to radiomics and genomics alone. These modest improvements may still be clinically meaningful in guiding NAC-related treatment decisions. ROC: Receiver operating characteristic; pCR: Pathological complete response; AUC: Area under the curve; CI: Confidence interval; NAC: Neoadjuvant chemotherapy

As depicted in Figure 2, these findings underscore the varying degrees of predictive accuracy among the three models, with the radiogenomic model standing out as the most effective in discerning patients who are likely to achieve pCR after NAC, although the differences were not statistically significant.

**Figure 3:**
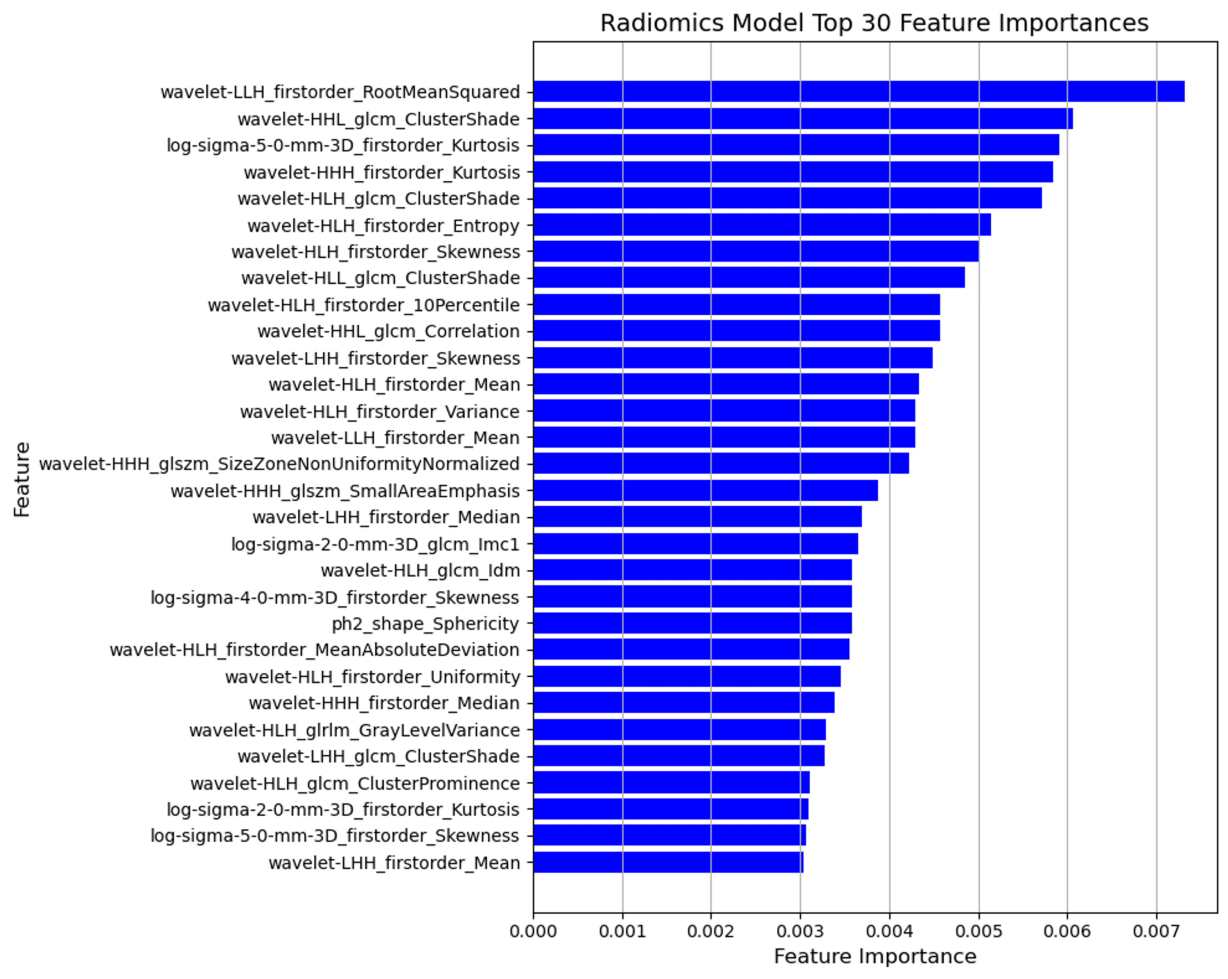
Top 30 feature importances for radiomics (MRI) model The bar plot displays the top 30 radiomic features extracted from MRI images.

**Figure 4:**
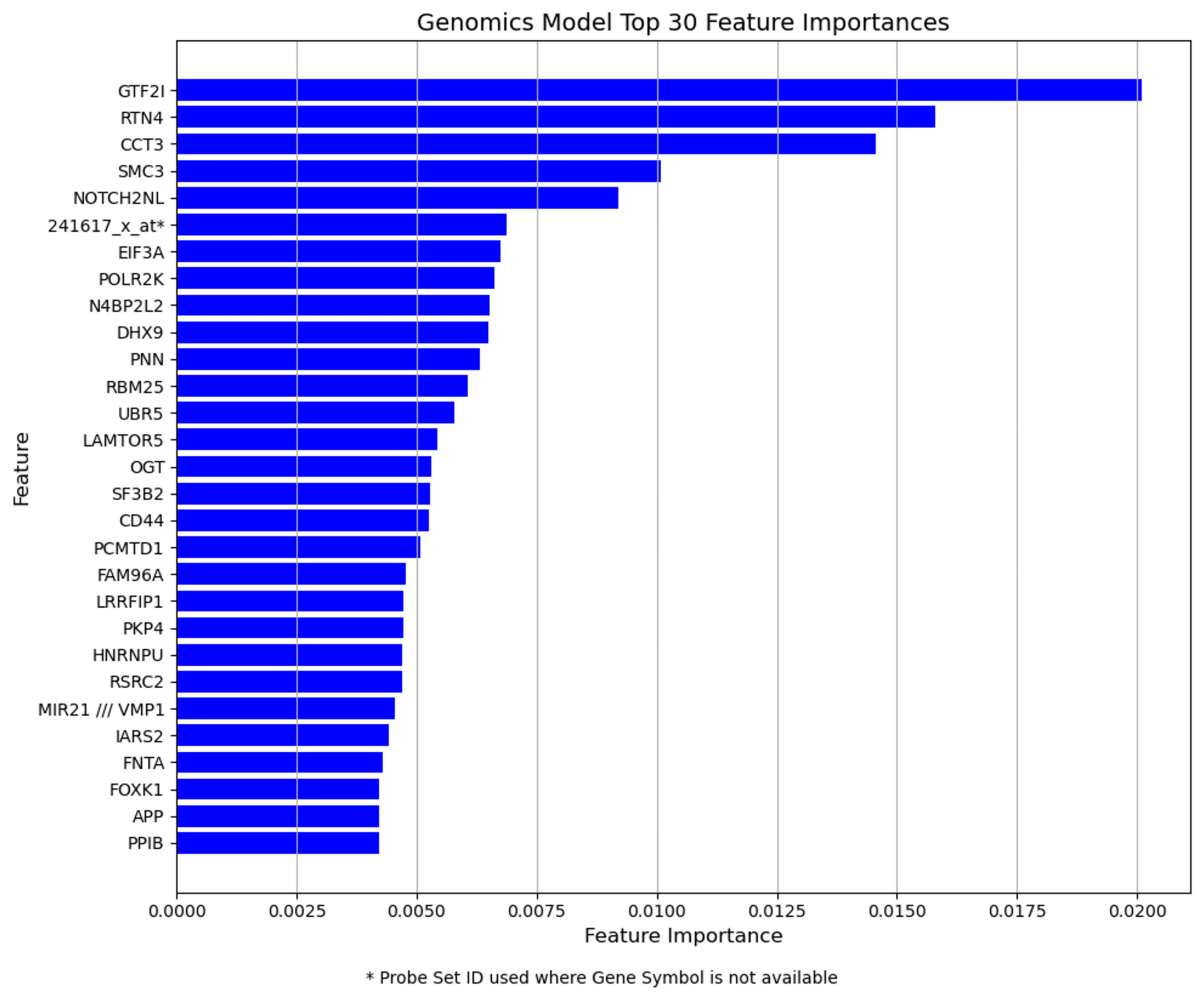
Top 30 feature importances for genomics (microarray) model The bar plot displays the top 30 genomic features based on DNA microarray gene expression data.

**Figure 5:**
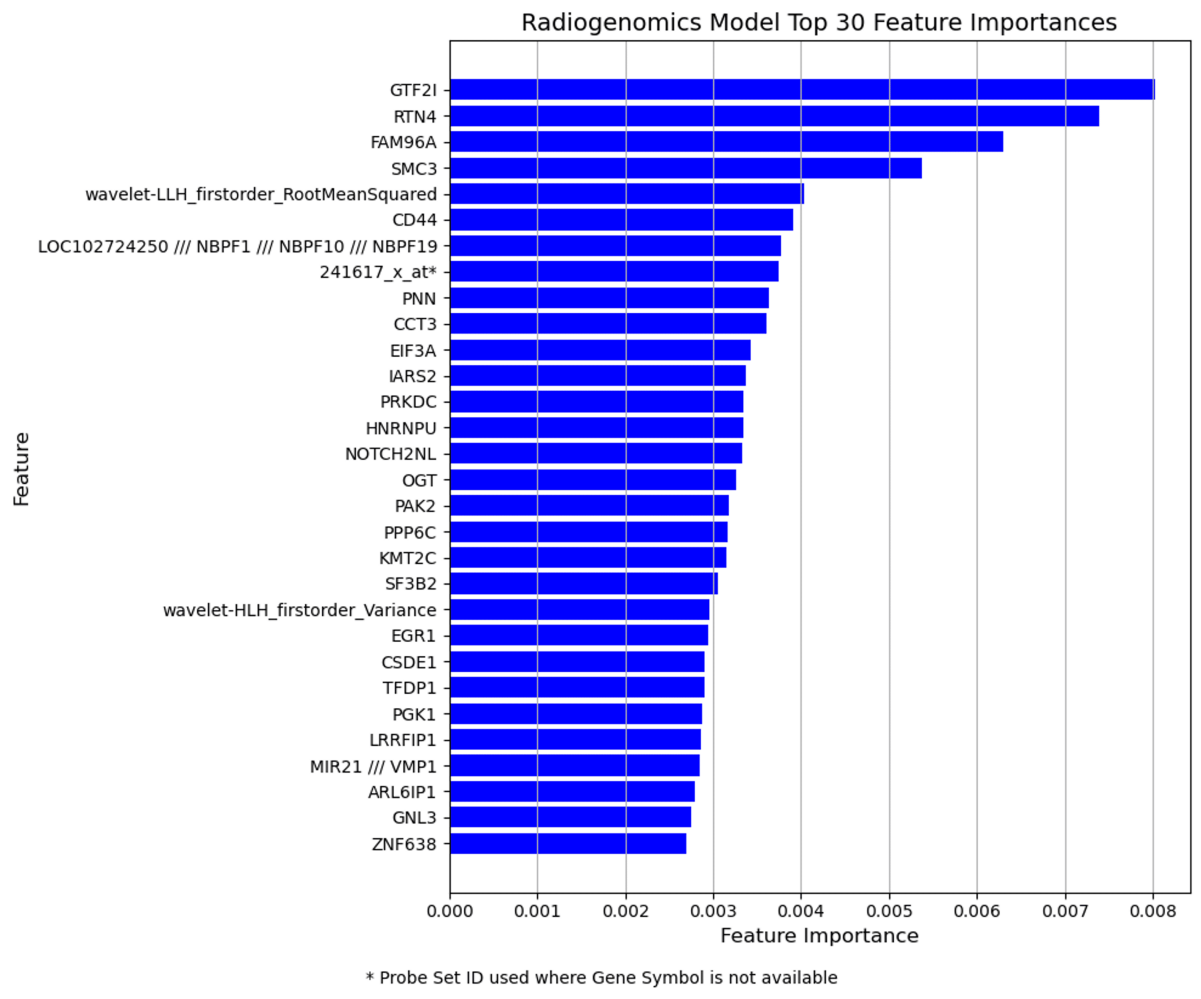
Top 30 feature importances for integrated radiogenomics (MRI+microarray) model The bar plot displays the top 30 features integrating MRI radiomics and DNA microarray gene expression data.

Figures 3, 4, 5 illustrate the feature importance scores derived from Random Forest classifiers for each predictive model. Each bar plot represents the top 30 features ranked by their mean decrease in impurity (Gini importance) scores, averaged across nested CV folds. Higher scores indicate greater importance of the features in predicting pCR. Gene symbols are shown where available; otherwise, Affymetrix probe set IDs are indicated (*).

## Discussion

In this study, we evaluated three machine-learning models, radiomics, genomics, and radiogenomics, to predict pCR after NAC in patients with breast cancer. The radiogenomics model, which combined MRI radiomic features with DNA microarray data, achieved the highest AUC of 0.607 (95% CI: 0.438-0.758) compared to the radiomics (AUC 0.563, 95% CI: 0.410-0.718) and genomics models (AUC 0.559, 95% CI: 0.379-0.722). Although the improvements were not statistically significant (p>0.05), these results suggest that integrating imaging data with genomic data may enhance the predictive accuracy of pCR.

Radiogenomics has demonstrated significant potential for improving early detection, prognosis, and treatment selection by integrating imaging features with genomic data, thereby advancing the field of personalized medicine [30]. Recent advances in radiogenomics have demonstrated the potential to improve the prediction of treatment responses in patients with breast cancer. Zhang et al. demonstrated the utility of a radiogenomic model in predicting pCR in patients with triple-negative breast cancer [25]. Zhou et al. developed a preoperative radiogenomic model based on quantitative heterogeneity that effectively predicted both pCR and prognosis in patients with triple-negative breast cancer undergoing NAC [26]. This was the first study to incorporate comprehensive gene expression data from microarrays into a radiogenomic model to enhance pCR prediction in all breast cancer subtypes. Our study provides new insights into the predictive performance of radiogenomics for pCR.

We constructed machine-learning models using MRI radiomic features and RNA expression profiles. These methods are consistent with the approach of Zheng et al., who integrated radiomic features with clinical characteristics to improve predictive accuracy [25]. Our study found that the radiogenomics model improved the performance of pCR prediction compared to the radiomics or genomics models. The analysis revealed that gene expression-related features were the top ones in the radiogenomic model, whereas MRI features also played an important role. These findings suggest that the combination of gene expression and MRI data contributes to improved pCR prediction.

First-order statistics and texture features processed using wavelet transforms and LoG filters are important in radiomics models. Features such as "wavelet-LLH_firstorder_RootMeanSquared" and "wavelet-HHL_glcm_ClusterShade" ranked highly. "wavelet-LLH_firstorder_RootMeanSquared" captures signal magnitude within wavelet-transformed images and reflects intratumoral heterogeneity, a known imaging correlate of poor treatment response. "wavelet-HHL_glcm_ClusterShade" quantifies textural asymmetry, potentially linked to microarchitectural irregularities and impaired drug delivery. These findings are consistent with prior radiomic studies highlighting the association between tumor heterogeneity and treatment resistance [12,26].

In a genomic model, the expression of genes involved in transcriptional regulation, protein synthesis, and chromosome maintenance is important. Features such as general transcription factor IIi (GTF2I), structural maintenance of chromosomes 3 (SMC3), and eukaryotic translation initiation factor 3, subunit A (EIF3A) ranked high. These proteins are known to play significant roles in cell proliferation, differentiation, and genome stability and are closely linked to cancer development and progression.

In the radiogenomic model, genetic features from microarray data were predominant. Moreover, the MRI-derived features are less important. The "wavelet-LLH_firstorder_RootMeanSquared" feature, which was the most important in the MRI model, ranked fifth in the combined model. This may be due to the greater predictive power and information density of the microarray data compared to MRI data. Gene expression data directly reflects the biological characteristics of cancer, potentially increasing their importance in the model.

Interestingly, some of the top features in the radiomic and genomic models remained in the radiogenomic model, suggesting their consistent predictive importance. Simultaneously, new top-ranking features emerged in the radiogenomic model, indicating that integrating data types could lead to new predictive factors. Furthermore, radiomic and genomic models represent two independent predictive frameworks for NAC sensitivity. By combining these models with a radiogenomic approach, we harnessed potentially complementary information that may enhance the accuracy of pCR prediction. Increasing the number of cases and further validating our findings using external cohorts are essential to establish the statistical significance and generalizability of this synergistic approach.

While further validation is needed, our findings suggest that radiogenomics may offer a practical framework for developing predictive tools that support personalized treatment planning. Integrating such models into clinical workflows could help identify patients more likely to benefit from NAC and avoid unnecessary treatment in those less likely to respond. As imaging and molecular profiling become increasingly accessible, combining these data sources may enhance clinical decision-making in breast cancer care. Moreover, the interpretability of the predictive models-supported by the use of biologically plausible features such as radiomic markers of tumor heterogeneity and genes involved in cell proliferation and structural regulation-may facilitate clinical acceptance and integration into future practice.

Our study has some limitations. First, this was a retrospective study conducted at a single institution. Second, the sample size was relatively small, which may have affected the robustness of the results and might have limited the ability to detect statistically significant differences between the predictive models. Nonetheless, even modest improvements in predictive performance may still be clinically meaningful in supporting treatment decisions for NAC in patients with breast cancer. Third, a potential selection bias existed, as the analysis was restricted to patients who received NAC and had available microarray data. Fourth, the segmentation method used for radiomic feature extraction may have influenced the results. As the annotations were manually performed, inter-observer variability may have affected the reproducibility of radiomic features. Although experienced readers were involved and standard annotation protocols were followed, some degree of variability is inevitable in manual segmentation. Fifth, no external validation was performed, which is important for confirming the reliability of the results. Sixth, the NAC regimens used in our study reflected the standards at the time of data collection, which may differ from current standard regimens. Finally, our analysis included a combination of chemotherapy and targeted therapy with only a small number of patients (N = 9/112, 8.0%) receiving trastuzumab. This could affect the relevance of our findings in contemporary clinical practice.

## Conclusions

This study demonstrated that radiogenomics, an integration of MRI-based radiomic features and gene expression profiles, showed the highest predictive performance for pCR following NAC in breast cancer. Although the improvement was not statistically significant compared to radiomics or genomics alone, the numerically superior performance of the radiogenomic model suggests that combining imaging and molecular data may enhance prediction accuracy in a non-invasive manner. Even modest improvements in predictive performance could be clinically meaningful in guiding individualized treatment decisions. These results support the potential of radiogenomics as a foundation for future, more personalized response prediction models in breast cancer management. However, to confirm its clinical utility, larger prospective studies involving diverse populations and external validation of the models will be essential.
